# Is the sustainable development goal target for financial risk protection in health realistic?

**DOI:** 10.1136/bmjgh-2016-000216

**Published:** 2017-09-28

**Authors:** Stéphane Verguet, Addis Tamire Woldemariam, Warren N Durrett, Ole F Norheim, Margaret E Kruk

**Affiliations:** 1 Department of Global Health and Population, Harvard T.H. Chan School of Public Health, Boston, Massachusetts, USA; 2 Federal Democratic Republic of Ethiopia, Ministry of Health, Addis Ababa, Ethiopia; 3 Department of Political Science, University of Washington, Seattle, Washington, USA; 4 Department of Global Public Health and Primary Care, University of Bergen, Bergen, Norway

**Keywords:** Sustainable Development Goals, financial risk protection, out-of-pocket costs, poverty, equity, low and middle-income countries

## Abstract

**Background:**

Setting Millennium Development Goals and Sustainable Development Goals for health has largely focused on defining specific targets of mortality and morbidity reduction over given time periods. Yet, less attention has been devoted to setting targets for the systemic determinants of health delivery, such as access and financial risk protection (FRP)—prevention of medical impoverishment. We examined candidate targets for FRP among low and middle-income countries by 2040.

**Methods:**

We used a data set on estimates of incidence of catastrophic health expenditure (CHE)—medical expenditure exceeding 40% of household capacity to pay—among 110 countries over 1995–2007, augmented by estimates of the percentage of out-of-pocket expenditure out of total health expenditure (OOP_EXP_), the share of health expenditure as a percentage of gross domestic product (HEX_GDP_) and the gross domestic product per capita (GDP_C_). Using a simple model and 2040 estimates for OOP_EXP_, HEX_GDP_ and GDP_C_ from the World Bank, the International Monetary Fund and the Institute for Health Metrics and Evaluation, we projected CHE incidence by 2040 for four country income groups.

**Results:**

We predicted that the 2040 incidence of CHE among households would be: 2.13% (Uncertainty interval: 0.60-6.87) among low-income countries, 1.15% (0.32–3.81) among lower-middle-income countries and 0.65% (0.18–2.21) among upper-middle-income countries. By 2040, the probability of achieving CHE <1.00% would be: 0.1 for low-income countries, 0.4 for lower-middle-income countries and 0.7 for upper-middle-income countries; for CHE <0.50%, it would be 0 for low-income countries, 0.1 for lower-middle-income countries and 0.3 for upper-middle-income countries.

**Conclusions:**

Historical trends of CHE rates can help define post-2015 targets for FRP. The projected achievements suggest that elimination of medical impoverishment will not be achieved by 2040 and that countries must urgently enact dramatic changes in policy to ensure FRP to their populations.

**Key questions**

**What is already known about this topic?**

- The Sustainable Development Goal for health (SDG3), ‘to ensure healthy lives and promote well-being for all at all ages,’ includes a subtarget on achieving ‘universal health coverage, including financial risk protection and access to quality, essential health services.’
- There has been little attention until recently to establishing an empirical basis for the measurement, tracking and setting of appropriate SDG targets for financial risk protection (FRP)—prevention of medical impoverishment—based on what might be possible to achieve in the future.

**What are the new findings?**

- We studied, as our measure of lack of FRP, observed past rates of catastrophic health expenditure—out-of-pocket medical expenditure exceeding 40% of household capacity to pay—and their determinants among countries over past times.
- We estimated the incidence of catastrophic health expenditure by country income group based on trajectories of health spending and financing, and subsequently tested candidate targets for FRP by the year 2040.
- Historical trends of catastrophic health expenditure rates can help define post-2015 targets for FRP. The projected achievements suggest that elimination of medical impoverishment would not be achieved by 2040.

## Introduction

The need to measure progress in health has been particularly apparent in relation to assessing whether countries are on track to achieve the Millennium Development Goals (MDGs).^1–3^ Measuring progress will also be crucial in determining whether countries can achieve the Sustainable Development Goals (SDGs)^4^ which were ratified by United Nations member states in September 2015 at the General Assembly in New York. The SDG for health (SDG3) is to ‘ensure healthy lives and promote well-being for all at all ages.’^5^ SDG3 also includes a subtarget (3.8) on achieving ‘universal health coverage, including financial risk protection and access to quality, essential health services.^5^

An important contribution of the universal health coverage (UHC) discussion is its focus on health-related financial burdens, particularly those due to payment for health services. One policy lever for reducing financial burden on families is to increase the proportion of prepayment (eg, social insurance, tax-based financing) and reducing out-of-pocket (OOP) financing in national health spending.^6^ Prepayment mechanisms and public finance may also lead to more efficient attainment of desired health outcomes.^7^

Attention to financial risk protection (FRP)—prevention of medical impoverishment—has grown in recent years as studies have found that OOP medical costs are a leading cause of impoverishment in many countries, and cross-country data have confirmed that high OOP health payments, often in the order of 40% and above of total health expenditure, increase risk of poverty.^8 9^ Expanding healthcare without FRP is not sustainable. Often, households choose from among several coping strategies in order to manage health-related expenses. When current income or savings are not sufficient, some resort to borrowing money or selling assets to pay for healthcare.^10^ Household medical expenditure can often be ‘catastrophic’^11 12^—defined as exceeding a certain fraction of total household expenditure or income.

While UHC has been extensively promoted in the past 5 years, there has been little attention until recently to establishing an empirical basis for the measurement, tracking and setting of appropriate targets for one of its essential components: FRP.^6 13–17^ One repeated statement has been that no households should face catastrophic medical expenditure from OOP payments,^16^ an aspiration which has been taken up in several global health areas such as tuberculosis (TB) (‘0% families facing catastrophic costs due to TB’)^18^ or surgery (‘100% protection against impoverishment from out-of-pocket payments for surgical and anaesthesia care’).^19^

Importantly, the WHO and the World Bank (WB) in their 2015 progress report towards UHC^17^ proposed to monitor the lack of FRP by two common indicators: the incidence of catastrophic health expenditure (CHE)—the proportion of households whose health expenditure is greater than a given threshold of total expenditure; and the incidence of impoverishment—the proportion of households pushed below the poverty line because of health expenditure. The WHO/WB report also included estimates on levels and trends of CHE for 37 countries over 2002–2012.^17^ This major effort, combined with a large mobilisation from the international and academic community, has led to refining the FRP measure within the SDGs^20^ with the inclusion of indicator 3.8.2: ‘lack of financial protection – proportion of the population with large household expenditure on health as a share of total household expenditure or income^21^

To the best of our knowledge, there has been no official SDG discussion on what could be FRP targets based on what might be possible to achieve in the future. In this respect, studying observed past rates of CHE—OOP medical expenditure exceeding 40% of household capacity to pay—and their determinants among countries over recent times might prove helpful in testing the feasibility of a proposal for a FRP target in the post-2015 agenda and enhancing accountability towards UHC while building on the WHO/WB framework. Hence, in this paper, we estimated the incidence of CHE by country income group based on trajectories of health spending and financing, and subsequently tested candidate targets for FRP by the year 2040 (a year for which estimated spending and financing data were available), relying on empirical and estimated data and using a rudimentary modelling approach.

## Methods

We chose the incidence of CHE, which is the percentage of households experiencing OOP medical expenditure greater than 40% of their capacity to pay, as defined by the WHO,^6^ as our measure of lack of FRP. Incidence of CHE was selected as our ‘dependent variable’ as readily useable estimates of CHE have been well documented and published for more than a hundred countries over 1995–2007,^9 22^ which we used in our analysis.

Two key determinants of the incidence of CHE at the country level identified by the seminal Xu and colleagues’ analysis^8^ were the percentage of OOP expenditure as of the total health expenditure and the percentage of health expenditure as a share of gross domestic product (GDP), which we denoted OOP_EXP_ and HEX_GDP_, respectively. We used OOP_EXP_ and HEX_GDP_ estimates as given by the WB^23^ for the time period 1995–2013 and as forecasted by the Institute for Health Metrics and Evaluation (IHME) and the WB^24^ for the year 2040. We also added GDP per capita, which we denoted GDP_C_. Wealthier countries offer more extensive healthcare services and their citizens have more resources to invest in health.

The list of all countries retained for the analysis is given in online supplementary appendix S1. Among the 179 countries, 32 were low income, 45 were lower middle income, 51 were upper middle income and 51 were high income, according to the WB’s income group classification.^23^

10.1136/bmjgh-2016-000216.supp1 Supplementary Appendix 1

First, following previous approaches,^8 9^ we estimated a simple model relating CHE to its key determinants of OOP_EXP_, HEX_GDP_ and GDP_C_ in the following way:

(1) $$\begin{array}{ll} \text{logit}(CHE)= & \beta_{0}+\beta_{1}\text{logit}(OOP_{EXP})+\beta_{2}\text{logit}(HEX_{GDP})\\ & +\beta_{3}\text{ln}(GDP_{c})+\beta_{r}+\varepsilon \end{array}$$

where $\beta_{r}$ is a country random effect capturing heterogeneity and variations between countries and studies and ε is an error term. A number of alternative model specifications could be tried and are discussed in online supplementary appendix S1. However, model (1) was retained for simplicity as data for OOP_EXP_, HEX_GDP_ and GDP_C_ (and not for other predictors) were readily available annually over 1995–2013, and recent estimates for OOP_EXP_ and HEX_GDP_ were available for the single year 2040.^24^

Second, OOP_EXP_ and HEX_GDP_ estimates were available for both the year 2013^23^ and the year 2040^24^; and GDP_C_ estimates were available for the year 2013.^23^ As for GDP_C_ estimates for 2040, from the projections for the years 2015–2021 from the International Monetary Fund,^25^ we calculated the annualised rates of change (in % per year) in GDP_C_ for low-income, lower-middle-income, upper-middle-income and high-income countries. Subsequently, we used such annualised rates of change to predict what would be GDP_C_, for each country, depending on their income group, for 2040. Using the 2040 values of GDP_C_, OOP_EXP_ and HEX_GDP_ in equation (1), we could estimate a predicted incidence of CHE by 2040 for each country, which was then aggregated per income group by arithmetic average.

Third, we compared our estimated 2040 incidence of CHE per income group to four candidate targets: (1) <0.50%; and three other targets, that is, (2) <1.00%; (3) <1.50%; and (4) <2.00%. To judge whether such targets might be realistic under current trends, we estimated the probability that each income group (low income; lower middle income; upper middle income) would have achieved them by 2040, according to our predictions.

### Uncertainty analysis

We pursued a probabilistic sensitivity analysis. For each income group (low income, lower middle income, upper middle income, high income), we pursued Monte Carlo simulations (n=100,000 trials) capturing both parameter uncertainty in the inputs (OOP_EXP_, HEX_GDP_, GDP_c_) and estimation uncertainty in the model. Parameter uncertainty was included through sampling *n* values for OOP_EXP_ and HEX_GDP_ parameters to which was assigned a beta distribution built on each input’s mean and uncertainty interval,^24^ and for the growth rate of GDP_c_ per income group to which was assigned a gamma distribution built on mean and SD.^25^ The estimation uncertainty was included through sampling *n* values for each combination of the model coefficients extracted from a multivariate Gaussian distribution using mean and variance-covariance matrices from the fitted model (1). Both parameter uncertainty and estimation uncertainty were varied simultaneously, resulting in *n* samples for each country CHE incidence. Through aggregation (arithmetic average) of country results within each income group, we again obtained *n* samples for CHE. Finally, extracting the 2.5 and 97.5 percentiles allowed the determination of 95% uncertainty intervals (UIs), which we reported along our results.

All analyses were conducted with the R statistical software version 3.3.1 (www.r-project.org).

## Results

We first report on the findings from model (1), where we observed that: for a relative reduction of 10% in the odds of OOP_EXP_ we obtained a relative reduction of 7% in the odds of CHE; for a relative increase of 10% in the odds of HEX_GDP_, we obtained a relative increase of 7% in the odds of CHE; and for a relative increase of 10% in GDP_C_, we obtained a relative decrease of 4% in the odds of CHE (table 1).

**Table 1:** Results for the predictors of logit of incidence of catastrophic health expenditure as a function of the percentage of OOP_EXP_, the percentage of HEX_GDP_ and the GDP_C_

Coefficient	Estimate	SE	p Value
logit(OOP_EXP_)	0.67	0.16	<0.001
logit(HEX_GDP_)	0.67	0.34	0.048
ln(GDP_C_)	−0.38	0.14	0.005

Goodness of fit, R^2^=0.69. Number of observations, 110. The variance of country random effects was 0.29.

GDP_C_, gross domestic product per capita; HEX_GDP_, health expenditure within the share of gross domestic product; OOP_EXP_, out-of-pocket expenditure within total health expenditure.

While examining the trends in GDP_C_ over 2015–2021, we calculated annual rates of change of 4.5% (SD 2.9%) for low-income countries, 4.5% (SD 2.7%) for lower-middle-income countries, 4.1% (SD 2.4%) for upper-middle-income countries and 3.4% (SD 2.0%) for high-income countries.

**Figure 1:**
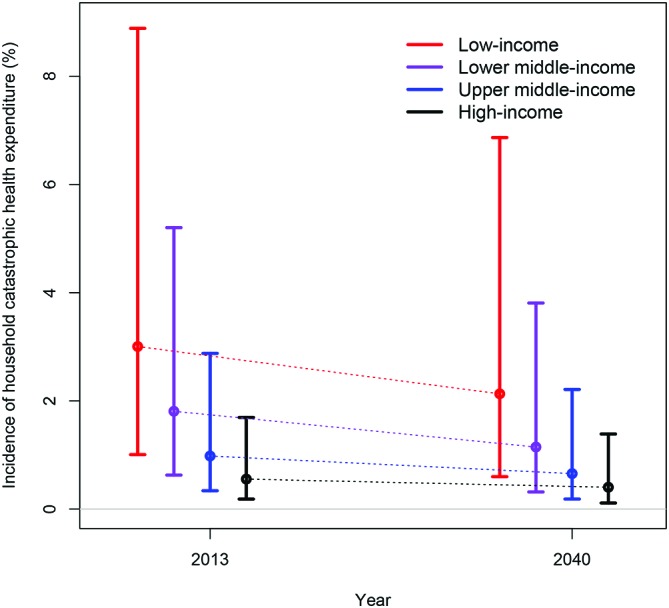
Estimated incidence and corresponding uncertainty ranges of catastrophic health expenditure among low-income (red), lower-middle-income (purple), upper-middle-income (blue) and high-income (black) countries in 2013 and 2040.

**Figure 2:**
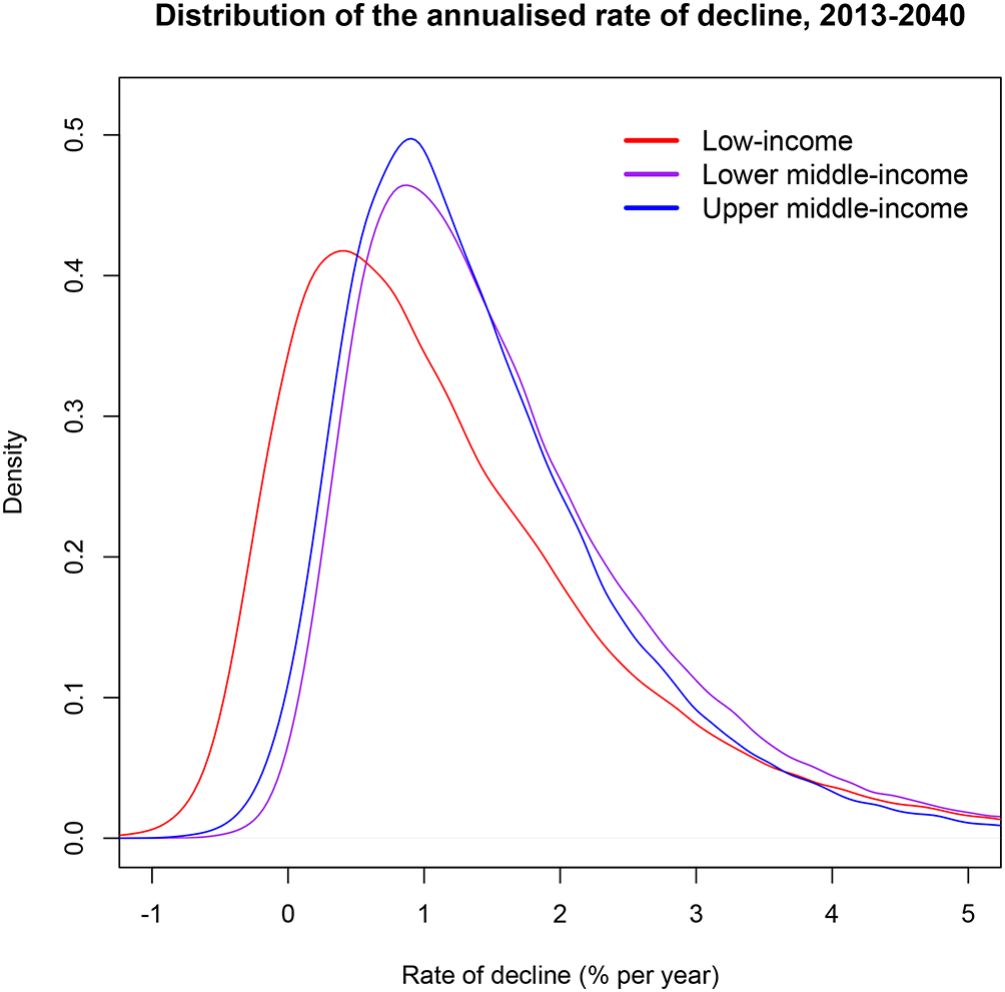
Distribution of the estimated annualised rate of decline over 2013–2040 of the incidence of catastrophic health expenditure among low-income (red), lower-middle-income (purple) and upper-middle-income (blue) countries.

With these inputs, we predicted the incidence of CHE among households in 2040 for each income group classification (figure 1). Estimated CHE would be: 2.13% (UI: 0.60-6.87)for low-income countries, 1.15% (0.32-3.81) for lower-middle-income countries, 0.65% (0.18-2.21) for upper-middle-income countries and 0.40% (0.11-1.39) for high-income countries. We also quantified the annualised rate of decline in the incidence of CHE over 2013–2040 (figure 2), which would be of: 1.3% (UI: −0.4% to 4.7%) per year for low-income countries, 1.7% (0.2%–4.8%) for lower-middle-income countries and 1.5% (0.0%–4.2%) for upper-middle-income countries.

**Figure 3:**
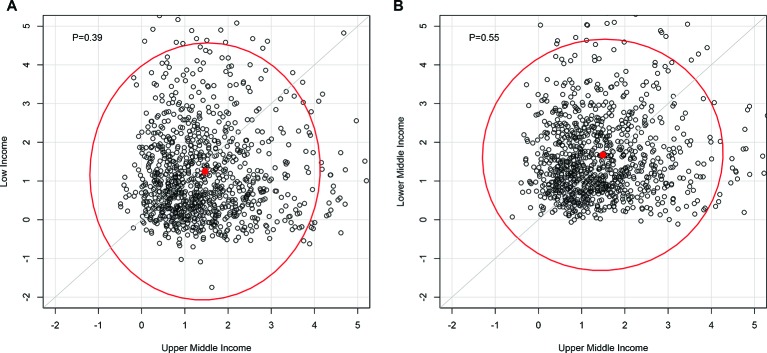
Rates of decline of incidence in catastrophic health expenditure over 2013–2040 of low-income (A) and lower-middle-income (B) countries (y-axis) compared with the rates of decline of upper-middle-income countries (x-axis) (n=1000 trials extracted from Monte Carlo simulations). A dot above the grey line indicates that the rate of decline of the income group is greater than the rate of decline of upper-middle-income countries; the corresponding probability is indicated in the top left corner and the 95% uncertainty contours in red.

Furthermore, we studied how the gaps in CHE incidence across income groups would evolve over 2013–2040 by examining whether the annualised rates of decline in CHE incidence (n=100,000) of the low-income and lower-middle-income countries group would be greater than those of the upper-middle-income countries group over the same time period (figure 3A,B). Notably, we found that low-income countries (probability of 0.39), compared with lower-middle-income countries (probability of 0.55), would be unlikely to reduce the gap with upper-middle-income countries.

No low-income countries would achieve CHE <0.50% by 2040 (table 2); the probability for lower-middle-income countries would be less than 0.10; the probability for upper-middle-income countries would be about 0.31. With a CHE target <1.0%, the probability for low-income countries would be 0.11, 0.39 for lower-middle-income countries and 0.74 for upper-middle-income countries. With a CHE target <1.5%, it would be: 0.27 for low-income countries, 0.65 for lower-middle-income countries and 0.90 for upper-middle-income countries.

**Table 2:** Probability (n=100,000 trials from Monte Carlo simulations) that country groups achieve specific financial risk protection targets of estimated incidence of CHE by 2040. Financial risk protection targets are: CHE <0.50%; CHE <1.00%; CHE <1.50%; and CHE <2.00%.

	CHE <0.50%	CHE <1.00%	CHE <1.50%	CHE <2.00%
Income group				
Low income	0.01	0.11	0.27	0.44
Lower middle income	0.09	0.39	0.65	0.80
Upper middle income	0.31	0.74	0.90	0.96

CHE, catastrophic health expenditure.

## Discussion

We presented in this paper estimates of the incidence of CHE in 2040 per country income group using readily available data and a simple modelling approach.

As identified previously,^8–10 15^ our analysis confirms the percentage of OOP expenditure out of total health expenditure (OOP_EXP_) as the main driver of CHE. According to our predictions, in 2040, the estimated incidence of CHE among households would be 2.13% (UI: 0.60-6.87) among low-income countries, 1.15% (0.31-3.81) among lower-middle-income countries and 0.65% (0.18-2.21) among upper-middle-income countries. The estimated relative reduction in CHE incidence over 2013–2040 would be of about 29% among low-income countries, 37% among lower-middle-income countries and 33% among upper-middle-income countries. This suggests that forecasts in financing policy would be supportive of FRP.

One purpose of our analysis is to help define what might be a feasible post-2015 target for FRP in health. In this respect, we found that, by 2040, the probability of achieving CHE <0.50% would be 0 for low-income countries, less than 0.10 for lower-middle-income countries and 0.31 for upper-middle-income countries. By contrast, with CHE <1.00%, it would be: 0.11 for low-income countries, 0.39 for lower-middle-income countries and 0.74 for upper-middle-income countries. While 0.50% and 1.0% both appear as low numbers, they translate into hundreds of thousands of people who are at risk of impoverishment if the reduction in OOP payments is not dramatically accelerated (1% of households correspond to about 2 500 000 families in India, 600 000 in Brazil and Indonesia, and 160 000 in Ethiopia, for example).

Our study has four key limitations. First and foremost, we relied on older CHE estimates^9 22^ which cover a time period over 1995–2007 and about a hundred and ten countries from all income levels. This points to the critical need that OOP health payments data be collected and reported routinely. New data on OOP payments are urgently needed and would be an important public good to support the global campaign for UHC. In this regard, the WHO/WB UHC tracking report^17^ critically provides directions and points to the limitations of past efforts including reliability and comparability across household expenditure surveys,^26–28^ and the needs for strengthening data availability and comparability in the future and for additional data notably on the long-term impact of household OOP payments and the distribution of those over time.^29^ Second, our modelling strategy was basic. More sophisticated approaches could have been used to forecast the changes in CHE incidence, and predict which countries would achieve distinct FRP targets by 2040. Here, we opted for a rudimentary  approach consistent with  previous work,^8 9^ which was readily applicable with underlying data (notably the available estimates for HEX_GDP_ and OOP_EXP_ for the year 2040^24^ and easily communicable to policymakers). Third, our study uniquely considered CHE, defined as medical expenditure exceeding 40% of household capacity to pay, as measure of (lack of) FRP. This measure was proposed as an indicator for monitoring progress towards UHC at country and global levels^16 17^: it is an indicator with which decision makers are familiar and for which data were readily available for analysis,^9 22^ hence our decision to use it in this study. Yet, the CHE metric presents a number of associated issues, most importantly the use of a defined threshold (ie, 40% of capacity to pay).^11^ Other metrics could be used such as impoverishing expenditure (eg, defined as medical expenditure pushing households under an absolute or relative poverty line), or forced borrowing or asset selling to finance medical expenditure.^10–12^ In addition, current CHE measures do not account for individuals who fail to obtain needed healthcare due to their inability to pay and thus are likely to underestimate financial burdens of illness. Finally, in Xu and colleagues^9^ three key determinants of high CHE were identified: high availability of health services, poverty and lack of prepayment mechanisms. Since countries differ substantially with respect to all three factors, their trajectory is extremely hard to predict. Due to data limitations, our model did not separate the availability of health services and shift towards prepayment mechanisms that accompanies income growth. If future expansion of health services availability is more rapid than the shift towards prepayments, CHE may well increase with economic growth. If we take into consideration the relative lack of adequate data, uncertainty and the dynamic interaction between these key determinants, we may conclude that lower CHE targets are more realistic, but still aspirational.

Protection from financial risks associated with healthcare expenses is a crucial component of national health strategies in many countries. From an ethical perspective, the loss of well-being from being pushed into poverty by health expenditure is no less devastating than the loss of health due to illness.^30^ Moving towards UHC is important because it will promote better health and it will protect people from poverty, the need to borrow money or possibly financial ruin. FRP is therefore a key objective for health systems.

This paper shows the reduction in CHE that may occur on current trajectory of health spending and financing policy.^24^ However, SDGs are aspirational by design. This analysis simply used historical trends and did not model what would result from increased priority to SDGs. Financing instruments including public finance and a higher share of public health expenditure through general taxation and payroll taxes could progressively replace OOP payments and reduce CHE. Countries spending more than 3% of public health expenditure as a share of GDP, as argued by *The Lancet* Commission on Investing in Health for achieving the ‘grand convergence’ around infectious, child and maternal mortality by 2035,^31^ often meet OOP_EXP_ under 20%.^32 33^ Using our model and setting OOP_EXP_ to a cap of 20% (or 15%) by 2040, the estimated incidence of CHE would become 0.86% (0.69) among low-income countries, 0.56% (0.47) among lower-middle-income countries and 0.45% (0.37) among upper-middle-income countries. This shows that large increases in public finance could substantially accelerate attainment of FRP. For instance, Thailand’s universal coverage scheme providing a comprehensive tax-financed benefits package given to the poor and those in the informal sector substantially reduced medical impoverishment since its inception in 2002.^34^ Likewise, the experience of several other low and middle-income countries suggests that UHC can hasten achievement of FRP.^35^ For example, through its Health Transformation Programme, Turkey substantially expanded insurance coverage within 10 years.^36^ With introducing *Seguro Popular* in 2003, Mexico has expanded access to comprehensive health services with FRP.^37^ Ethiopia is currently maintaining selected essential services free of charge and rolling out health insurance schemes on its way to UHC (box).

Box **Provision of financial risk protection in Ethiopia**

Ethiopia has long recognised that direct out-of-pocket (OOP) payments at the point of care prevent a majority of the population from accessing health services and result in financial hardship for many, and has therefore designed two appropriate strategies: maintaining selected high-impact fee-exempted services for everyone, and risk pooling and prepayment mechanisms.

The first strategy is putting in place a fee exemption for a set of selected high-impact and cost-effective services including vaccinations, in-facility birth services, antiretroviral treatment, tuberculosis treatment, and so on. Such ‘exempted’ essential services organised into the five categories of family health, communicable disease prevention and control, hygiene and environmental health, health education and communication, basic curative care and treatment of chronic conditions, have been provided to all free of charge and will remain so.^38^

Second, there are two insurance schemes. Social health insurance (SHI) is a compulsory scheme for people in the formal sector where employees and employers contribute to the scheme, and which has about 1.8 million members. Community-based health insurance (CBHI) is a voluntary scheme meant for those in the informal sector and is largely subsidised by the government. It has been successfully scaled up in 200 districts (25% of Ethiopia’s districts).^39^ The next 5-year health sector plan, the Health Sector Transformation Plan (HSTP), sets 100% coverage for SHI and 80% for CBHI, and puts targets of reducing OOP expenditure from 34% to 15% of total health expenditure by 2020.^40 41^ The primary objective is to reduce the reliance on direct OOP payments, lower the financial barriers to access and thereby diminish the impoverishing impact. With these two schemes, the level and share of revenues channelled through prepaid and pooled mechanisms will be increased, reducing fragmentation to increase the redistributional capacity of the pooled funds, on the move towards universal coverage. In the long term, the HSTP intends to harmonise the benefits packages of and integrate the two schemes.

To achieve substantial improvements in FRP in the decades ahead, dramatic policy changes need to happen in low and middle-income countries, led by countries and supported by the international community. Thus, our work here should be viewed as a conservative estimate of what the future holds. The purpose of UHC and, indeed of the SDGs, is to change the future.

## References

[1] Bhutta ZA, Chopra M, Axelson H, et al Countdown to 2015 decade report (2000-10): taking stock of maternal, newborn, and child survival. Lancet 2010;375:2032–44. 10.1016/S0140-6736(10)60678-2 20569843

[2] Wang H, Liddell CA, Coates MM, et al Global, regional, and national levels of neonatal, infant, and under-5 mortality during 1990-2013: a systematic analysis for the global burden of disease study 2013. Lancet 2014;384:957–79. 10.1016/S0140-6736(14)60497-9 24797572 PMC4165626

[3] Kassebaum NJ, Bertozzi-Villa A, Coggeshall MS, et al Global, regional, and national levels and causes of maternal mortality during 1990-2013: a systematic analysis for the global burden of disease study 2013. Lancet 2014;384:980–1004. 10.1016/S0140-6736(14)60696-6 24797575 PMC4255481

[4] Verguet S, Norheim OF, Olson ZD, et al Annual rates of decline in child, maternal, HIV, and tuberculosis mortality across 109 countries of low and middle income from 1990 to 2013: an assessment of the feasibility of post-2015 goals. Lancet Glob Health 2014;2:e698–e709. 10.1016/S2214-109X(14)70316-X 25433625

[5] United Nations. https://sustainabledevelopment.un.org/ (accessed Jan 18 2016).

[6] World Health Organization. 2010 World Health Report 2010. Health Systems Financing, The Path to Universal Coverage. Geneva: World Health Organization. 10.2471/BLT.10.078741 PMC2878164 20539847

[7] Moreno-Serra R, Smith PC Does progress towards universal health coverage improve population health? Lancet 2012;380:917–23. 10.1016/S0140-6736(12)61039-3 22959388

[8] Xu K, Evans DB, Kawabata K, et al Household catastrophic health expenditure: a multicountry analysis. Lancet 2003;362:111–7. 10.1016/S0140-6736(03)13861-5 12867110

[9] Xu K, Evans DB, Carrin G, et al Protecting households from catastrophic health spending. Health Aff 2007;26:972–83. 10.1377/hlthaff.26.4.972 17630440

[10] Kruk ME, Goldmann E, Galea S Borrowing and selling to pay for health care in low- and middle-income countries. Health Aff 2009;28:1056–66. 10.1377/hlthaff.28.4.1056 19597204

[11] Wagstaff A Measuring financial protection in health : Smith PC, Mossialos E, Papanicolas I Leatherman S, Performance measurement for health system improvement. Cambridge, UK: Cambridge University Press, 2010.

[12] Wagstaff A, van Doorslaer E Catastrophe and impoverishment in paying for health care: with applications to Vietnam 1993-1998. Health Econ 2003;12:921–33. 10.1002/hec.776 14601155

[13] Frenk J, de Ferranti D Universal health coverage: good health, good economics. Lancet 2012;380:862–4. 10.1016/S0140-6736(12)61341-5 22959372

[14] Kruk ME Universal health coverage: a policy whose time has come. BMJ 2013;347:f6360 10.1136/bmj.f6360 24153242

[15] Saksena P, Hsu J, Evans DB Financial risk protection and universal health coverage: evidence and measurement challenges. PLoS Med 2014;11:e1001701 10.1371/journal.pmed.1001701 25244520 PMC4171370

[16] Boerma T, Eozenou P, Evans D, et al Monitoring progress towards universal health coverage at country and global levels. PLoS Med 2014;11:e1001731 10.1371/journal.pmed.1001731 25243899 PMC4171369

[17] World Health Organization, World Bank. Tracking Universal Health Coverage – First Global Monitoring Report. Geneva: World Health Organization, 2015.

[18] WHO. Introducing the end TB strategy. Geneva: World Health Organization, 2015 http://www.who.int/tb/End_TB_brochure.pdf (accessed 18 Jan 2016).

[19] Meara JG, Leather AJ, Hagander L, et al Global Surgery 2030: evidence and solutions for achieving health, welfare, and economic development. Lancet 2015;386:569–624. 10.1016/S0140-6736(15)60160-X 25924834

[20] McIntyre D, McKee M, Balabanova D, et al Open letter on the SDGs: a robust measure for universal health coverage is essential. Lancet 2016;388:2871–2. 10.1016/S0140-6736(16)32189-4 27863812

[21] WHO. Monitoring sustainable development goals. http://www.who.int/health_financing/topics/financial-protection/monitoring-sdg/en/ (accessed 18 Mar 2017).

[22] Xu K, Saksena P, Jowett M et al Exploring the Thresholds of Health Expenditure for Protection Against Financial Risk. World Health Report 2010 Background Paper 19. Geneva: World Health Organization, 2010.

[23] World Bank. World development indicators. http://data.worldbank.org/data-catalog/world-development-indicators (accessed 22 Mar 2017).

[24] Dieleman JL, Templin T, Sadat N, et al National spending on health by source for 184 countries between 2013 and 2040. Lancet 2016;387:2521–35. 10.1016/S0140-6736(16)30167-2 27086174

[25] International Monetary Fund. 2016 World Economic Outlook Database. Washington, DC: International Monetary Fund.

[26] Xu K, Ravndal F, Evans DB, et al Assessing the reliability of household expenditure data: results of the World Health Survey. Health Policy 2009;91:297–305. 10.1016/j.healthpol.2009.01.002 19217184

[27] Heijink R, Xu K, Saksena P, et al Validity and comparability of out-of-pocket health expenditure from household surveys: a review of the literature and current survey instruments. Geneva: World Health Organization, 2011 http://www.who.int/health_financing/documents/dp_e_11_01-oop_errors.pdf (accessed 19 Mar 2017).

[28] Lavado RF, Brooks BP, Hanlon M Estimating health expenditure shares from household surveys. Bull World Health Organ 2013;91:519–24. 10.2471/BLT.12.115535 23825879 PMC3699797

[29] Flores G, Krishnakumar J, O’Donnell O, et al Coping with health-care costs: implications for the measurement of catastrophic expenditures and poverty. Health Econ 2008;17:1393–412. 10.1002/hec.1338 18246595

[30] World Health Organization. Making fair choices on the path to universal health coverage. Geneva: World Health Organization, 2014. 10.2471/BLT.14.139139 PMC4047814 24940009

[31] Jamison DT, Summers LH, Alleyne G, et al Global health 2035: a world converging within a generation. Lancet 2013;382:1898–955. 10.1016/S0140-6736(13)62105-4 24309475

[32] Beattie A, Yates R, Noble DJ Accelerating progress towards universal health coverage in Asia and Pacific: improving the future for women and children. BMJ Glob Health 2016;1(Suppl 2):i12–i18. 10.1136/bmjgh-2016-000190 PMC5418650 28588989

[33] Beattie A, Yates R, Noble D Accelerating progress towards universal health coverage for women and children in South Asia, East Asia and the Pacific. UNICEF and Chatham House, 2016. http://billion-brains.org/wp-content/uploads/2016/10/UHC-Paper.pdf (accessed 17 Mar2017). 10.1136/bmjgh-2016-000190 PMC5418650 28588989

[34] Tangcharoensathien V, Limwattananon S, Patcharanarumol W, et al Monitoring and evaluating progress towards Universal Health Coverage in Thailand. PLoS Med 2014;11:e1001726 10.1371/journal.pmed.1001726 25243409 PMC4171094

[35] Lagomarsino G, Garabrant A, Adyas A, et al Moving towards universal health coverage: health insurance reforms in nine developing countries in Africa and Asia. Lancet 2012;380:933–43. 10.1016/S0140-6736(12)61147-7 22959390

[36] Atun R, Aydın S, Chakraborty S, et al Universal health coverage in Turkey: enhancement of equity. Lancet 2013;382:65–99. 10.1016/S0140-6736(13)61051-X 23810020

[37] Knaul FM, González-Pier E, Gómez-Dantés O, et al The quest for universal health coverage: achieving social protection for all in Mexico. Lancet 2012;380:1259–79. 10.1016/S0140-6736(12)61068-X 22901864

[38] Ministry of Health of Ethiopia. The Revised National Health Policy of the Federal Democratic Republic of Ethiopia. Addis Ababa: Ministry of Health of Ethiopia, 2015.

[39] Ethiopian Health Insurance Agency. Evaluation of Community-Based Health Insurance Pilot Schemes in Ethiopia: Final Report. Addis Ababa, Ethiopia, 2015.

[40] Ministry of Health of Ethiopia. Health Sector Transformation Plan 2016-2020. Addis Ababa: Ministry of Health of Ethiopia, 2015.

[41] Ministry of Health of Ethiopia. Annual Performance Report of the Health Sector 2014. Addis Ababa: Ministry of Health of Ethiopia, 2014.

