# Supplementary material for: Is the sustainable development goal target for financial risk protection in health realistic?
Source: BMJ Glob Health. 2017 Sep 28;2(3):e000216. doi: 10.1136/bmjgh-2016-000216 (PMC5639981; doi:10.1136/bmjgh-2016-000216)
Supplement: Supplementary Appendix 1 [file bmjgh-2016-000216supp001.pdf]

Supplementary web appendix

Web appendix I – List of countries included in the analysis

There are 179 low- and middle-income countries included in the analysis.[1]

The 32 low-income countries are: Afghanistan, Bangladesh, Burundi, Benin, Burkina Faso, Central African Republic, Comoros, Ethiopia, Guinea, The Gambia, Guinea-Bissau, Haiti, Cambodia, Kenya, Kyrgyzstan, Liberia, Madagascar, Mali, Mozambique, Myanmar, Malawi, Niger, Nepal, Rwanda, Sierra Leone, South Sudan, Chad, Tajikistan, Togo, Tanzania, Uganda, the Democratic Republic of the Congo.

The 45 lower-middle-income countries are: Armenia, Bhutan, Bolivia, Cameroon, Cape Verde, Congo, Côte d'Ivoire, Djibouti, Egypt, El Salvador, Micronesia, Georgia, Ghana, Guatemala, Guyana, Honduras, India, Indonesia, Kiribati, Laos, Lesotho, Mauritania, Moldova, Mongolia, Morocco, Nicaragua, Nigeria, Pakistan, Papua New Guinea, Paraguay, Philippines, Samoa, Sao Tome and Principe, Senegal, Solomon Islands, Sri Lanka, Sudan, Swaziland, Timor-Leste, Ukraine, Uzbekistan, Vanuatu, Vietnam, Yemen, Zambia.

The 51 upper-middle-income countries are: Albania, Algeria, Angola, Argentina, Azerbaijan, Belarus, Belize, Bosnia and Herzegovina, Botswana, Brazil, Bulgaria, China, Colombia, Costa Rica, Cuba, Dominica, Dominican Republic, Ecuador, Fiji, Gabon, Grenada, Hungary, Iran, Iraq, Jamaica, Jordan, Kazakhstan, Lebanon, Macedonia, Malaysia, Maldives, Marshall Islands, Mauritius, Mexico, Montenegro, Namibia, Panama, Peru, Romania, Saint Lucia, Saint Vincent and the Grenadines, Serbia, Seychelles, South Africa, Suriname, Thailand, Tonga, Tunisia, Turkey, Turkmenistan, Venezuela.

The 51 high-income countries are: Antigua and Barbuda, Australia, Austria, Bahrain, Barbados, Belgium, Brunei, Canada, Chile, Croatia, Cyprus, Czech Republic, Denmark, Equatorial Guinea, Estonia, Finland, France, Germany, Greece, Iceland, Ireland, Israel, Italy, Japan, Kuwait, Latvia, Lithuania, Luxembourg, Malta, Netherlands, New Zealand, Norway, Oman, Poland, Portugal, Qatar, Russia, Saudi Arabia, Singapore, Slovakia, Slovenia, South Korea, Spain, Sweden, Switzerland, the Bahamas, Trinidad and Tobago, United Arab Emirates, United Kingdom, United States of America, Uruguay.

The data inputs for incidence of catastrophic health expenditure were extracted from Xu et al.'s supplementary appendix[2,3] and included the following 110 countries and years: Albania (1996), Argentina (1996/97, 2002), Armenia (1998/99, 2001), Austria (1999/00), Azerbaijan (1995), Bangladesh (1995/96), Belarus (1999), Belgium (1997/98), Bolivia (1999, 2000, 2001, 2002), Brazil (1996), Bulgaria (1995, 1997, 2000), Burkina Faso (1998), Cambodia (1999), Canada (1997), Cape Verde (2001/02), China (2000), Colombia (1997), Côte d'Ivoire (1998), Croatia (1999), Denmark (1997), Djibouti (1996), Egypt (1997), Estonia (1995, 2001, 2002, 2005), Finland (1998), France (1995, 2006), Georgia (1999, 2007), Ghana (1998/99), Greece (1998), Hungary (2000), Iceland (1995), India (1995), Indonesia (1999, 2000, 2001), Ireland (1999/00), Israel (1999), Italy (2001), Jamaica (1997, 2001), Kazakhstan (1996), Kenya (2003), Kyrgyzstan (1998, 2004), Laos (1997/98), Latvia (1997/98, 2006), Lebanon (1999), Lithuania (1999), Malawi (1997/98, 2003), Malaysia (1998/99), Mauritius (1996/97), Mexico (1996), Moldova (2007), Morocco (1998/99), Nepal (1995/96), Nicaragua (1998, 2001), Norway (1996, 1997, 1998), Oman (1999/00), Panama (1997), Paraguay (1996, 2000/01), Peru (2000), Philippines (1997), Portugal (1994/95), South Korea (1999, 2007), Russia (1998, 2000, 2002), Rwanda (2005/06), Senegal (1994/95), Serbia-Montenegro (2000), Slovenia (1997/98), South Africa

(1995), Spain (1996), Sri Lanka (1995/96), Sweden (1996), Switzerland (1998), Tajikistan (1999), Thailand (1996, 1998), Tunisia (1995), Uganda (1996/97, 1999/00, 2002/03), Ukraine (1996, 2006), United Kingdom (1999/00), Uruguay (1997), United States (1995), Vietnam (1997), Yemen (1998), Zambia (1996). The years prior to 1995 were not included as data for the independent variables (e.g. OOP_{EXP}, HEX_{GDP}) were only available from 1995 onwards.[1]

References

1. World Bank. World development indicators. Available from: http://data.worldbank.org/data-catalog/world-development-indicators (accessed August 21, 2015).
2. Xu K, Evans DB, Carrin G, et al. Protecting households from catastrophic health spending. Health Affairs 2007; 6(4):972-983.
3. Xu K, Saksena P, Jowett M, Indikadah C, Kutzin J, Evans D. Exploring the thresholds of health expenditure for protection against financial risk. World Health Report 2010 Background paper 19. Geneva: World Health Organization, 2010.

Web appendix II – Alternative model specifications

We conducted a range of linear regression analyses including a variety of independent variables to examine alternative specifications to the linear model (1) used in the main text.

These independent variables included: the poverty headcount ratio at \$1.25 a day (purchasing power parity, PPP) (% of population),[1] the poverty headcount ratio at national poverty lines (% of population),[1] the Gini index,[1] the mean years of education for both sexes for those aged 15 and above,[2] health expenditure per capita, PPP (constant 2011 international \$).[1] The results are collected in table S1.

An alternative to HEX_{GDP} could be public health expenditure as a percentage of GDP, denoted $PHEX_{GDP}$, and the model (1) (main text) used in our analysis could be adapted in the following:

$$\text{logit}(CHE) = \beta_0 + \beta_1 \,\text{logit}(OOP_{EXP}) + \beta_2 \,\text{logit}(PHEX_{GDP}) + \beta_3 \ln(GDP_C) + \beta_r + \varepsilon\,, \quad \text{(S1)}$$

where β_r is a country random effect capturing heterogeneity and variations between countries and studies and ε is an error term. $PHEX_{GDP}$ was also available for the year 2013[1] and could be estimated for the year 2040.[3] The regression results from model (S1) are reported in table S2.

However, we selected model (1) (main text) as OOP_{EXP} and HEX_{GDP} were two key determinants of CHE identified by the work of Xu and colleagues,[4,5] to which we intend to be consistent with. In this seminal work,[4] three determinants of CHE were exhibited: availability of health services, low capacity to pay that can be proxied by GDP_C, and the lack of pre-payment mechanisms that can be proxied by OOP_{EXP}. As indicated by Xu et al.[4] HEX_{GDP} can proxy the availability of health services: the more services being used for a given level of GDP, the more likely households are to face CHE. Furthermore, OOP_{EXP} is a complement to pre-payment mechanisms, which is funded mainly through public finance related to $PHEX_{GDP}$. Indeed,

OOP_{EXP} and $PHEX_{GDP}$ are highly negatively correlated (e.g. -0.62 for the year 2013); in addition, $PHEX_{GDP}$ and GDP_C are positively correlated (e.g. 0.30 for the year 2013), hence our choice to use model (1) and HEX_{GDP}.

Table S1. Results of models predicting catastrophic health expenditure (logit) in low- and middle-income countries.

logit(OOP_{EXP})	0.67 (0.16)	0.64 (0.17)	0.68 (0.17)	0.67 (0.18)	0.67 (0.16)	0.67 (0.16)
logit(HEX_{GDP})	0.67 (0.34)	0.59 (0.36)	0.56 (0.34)	0.54 (0.40)	0.13 (0.65)	0.63 (0.32)
ln(GDP_C) (2011 international $)	-0.38 (0.14)	-0.35 (0.15)	-0.53 (0.20)	-0.42 (0.20)	-0.93 (0.58)	-0.63 (0.15)
ln(Gini)		1.07 (0.53)				
Pov (Int$1.25 per day)			-0.01 (0.01)			
Pov (National)				0.00 (0.01)		
ln(Hcap) (2011 international $)					0.58 (0.60)	
Edu						0.02 (0.01)
Number of observations	110	104	107	93	110	106
Goodness of fit R^2	0.69	0.66	0.66	0.65	0.71	0.67

Standard errors in parentheses.
OOP_{EXP} = share (%) of total health expenditure borne out-of-pocket; HEX_{GDP} = total health expenditure as a percent of gross domestic product; GDP_C = gross domestic product per capita, in 2011 international $; Pov = poverty headcount ratio (% of population) at either $1.25 a day Purchasing Power Parity or national poverty lines; Gini = Gini index; Hcap = health expenditure per capita, in 2011 international $; Edu = mean years of education for both sexes for those aged 15 and above.

Table S2. Results of the model predicting logit of incidence of catastrophic health expenditure, as a function of the percentage of out-of-pocket expenditure within total health expenditure (OOP_{EXP}), the percentage of public health expenditure within the share of gross domestic product ($PHEX_{GDP}$), and the gross domestic product per capita (GDP_C).

Coefficient	Estimate	Standard error	P - value
logit(OOP_{EXP})	1·14	0·21	< 0·001
logit($PHEX_{GDP}$)	1·24	0·34	< 0·001
ln(GDP_C)	-0·49	0.14	< 0·001

Goodness of fit: R^2 = 0·72. Number of observations: 110. The variance of country random effects was 0·26.

References

1. World Bank. World development indicators. Available from: http://data.worldbank.org/data-catalog/world-development-indicators (accessed August 21, 2015).
2. Institute for Health Metrics and Evaluation (IHME). Global educational attainment 1970-2015. Seattle, United States: Institute for Health Metrics and Evaluation (IHME), 2015. Available from: http://ghdx.healthdata.org/record/global-educational-attainment-1970-2015 (accessed August 29, 2015).
3. Dieleman J, Templin T, Sadat N, Reidy P, Chapin A, et al. National spending on health by source for 184 countries between 2013 and 2040. Lancet 2016; 387:2521-2535.
4. Xu K, Evans DB, Kawabata K, et al. Household catastrophic health expenditure: a multicountry analysis. Lancet 2003; 362:111-117.
5. Xu K, Evans DB, Carrin G, et al. Protecting households from catastrophic health spending. Health Affairs 2007; 6(4):972-983.
